# Effects of primary care cost-sharing among young adults: varying impact across income groups and gender

**DOI:** 10.1007/s10198-019-01095-6

**Published:** 2019-08-10

**Authors:** Naimi Johansson, Niklas Jakobsson, Mikael Svensson

**Affiliations:** 1 Health Metrics, Sahlgrenska Academy at University of Gothenburg, PO Box 463, 405 30 Gothenburg, Sweden; 2 Department of Economics, Karlstad University, Universitetsgatan 2, 651 88 Karlstad, Sweden; 3 Norwegian Social Research (NOVA), Oslo, Norway

**Keywords:** Patient cost-sharing, Health care demand, Price sensitivity, Income inequality, D12, H51, I11, I18

## Abstract

We estimate the price sensitivity in health care among adolescents and young adults, and assess how it varies across income groups and gender, using a regression discontinuity design. We use the age differential cost-sharing in Swedish primary care as our identification strategy. At the 20th birthday, the copayment increases from €0 to approx. €10 per primary care physician visit and close to this threshold the copayment faced by each person is distributed almost as good as if randomized. The analysis is performed using high-quality health care and economic register data of 73,000 individuals aged 18–22. Our results show that the copayment decreases the average number of visits by 7%. Among women visits are reduced by 9%, for low-income individuals by 11%, and for low-income women by 14%. In conclusion, modest copayments have significant utilization effects, and even in a policy context with relatively low income inequalities, the effect is substantially larger in low-income groups and among women.

**Electronic supplementary material:**

The online version of this article (10.1007/s10198-019-01095-6) contains supplementary material, which is available to authorized users.

## Introduction

Patient cost-sharing in health care is a standard policy instrument to reduce the risk of moral hazard and to increase efficiency [cf. 1]. However, a concern with cost-sharing is that it may reduce compliance with relevant care, and thus cause negative health consequences [2, 3]. Also, if cost-sharing has a larger impact on low-income households, as suggested by some authors [e.g. 2], it will increase income-related inequalities in health care utilization.

To assess the welfare and (in)equality consequences of cost-sharing, we need evidence on the behavioral responses to cost-sharing and potential differences across demographic and socio-economic groups. Although reviews have identified more than 50 papers that have analyzed utilization effects of out-of-pocket prices [4, 5], only a small share of these papers is based on experimental or quasi-experimental research designs that can credibly claim to address the endogeneity issues (e.g., the price a patient faces is not random). Papers with strong research designs include, e.g., the Rand Health Insurance Experiment [e.g., 6], which indicated a price elasticity of demand at around − 0.2 [7]. More recently, Chandra et al. [8] utilized exogenous variation in copayments faced by low-income groups in Massachusetts and estimated an average price elasticity of − 0.16. Analyzing data from the Oregon Medicaid lottery, a significant increase in utilization was seen for low-income groups upon receiving Medicaid coverage [9].

Non-US evidence include, for example, regression discontinuity (RD) design studies in Japanese and Taiwanese contexts with elasticity estimates around − 0.1 to − 0.2 [10–12]. Recent evidence of children’s and adolescents’ demand for medical care using Swedish data find differential effects based on family income, where children of low-income households respond stronger to a copayment increase [13]. In sum, most studies find significant but relatively modest price sensitivity.

Very few of the mentioned papers are able to directly compare the price elasticity across income groups, and instead rely on indirect comparisons based on estimates from different populations in different contexts. Our main contribution to the literature is that we are able to make direct comparisons across the full range of income groups using high-quality register data. Compared to Nilsson and Paul [13], who apply a similar empirical strategy, we contribute with recent and more detailed data, and additionally provide an extensive set of robustness checks to demonstrate the validity of our empirical method. Our study design is based on regression discontinuity (RD) analyses which use the fact that in Swedish primary care, there is a discontinuity in the copayment scheme at the age of 20. Up until a person turns 20 there is no copayment, at the 20th birthday and beyond, the copayment is 100 Swedish kronor (SEK) per physician visit (SEK 100 ≈ €10 in 2018). The Swedish policy context is one with relatively modest economy-wide income inequalities, with a GINI index of 29.2 compared to, e.g., 41.5 in the US [14]. Thus, we could expect the variation in price elasticity across income groups to be smaller in Sweden and potentially seen as lower-bound estimates in an international perspective.

Further, we contribute to the emerging literature on forward-looking behavior in health care policy. An additional complication in papers that use quasi-experimental research designs based on policy reforms or prices that vary by age, is that observed effects on utilization may be caused by a pure price effect at some point in time and/or by an anticipation effect due to forward-looking behavior. Assuming that individuals are forward-looking, they are likely to adapt to known changes in future policy [15, 16]. For example, evidence from the US suggests postponement of certain medical care awaiting Medicare coverage at age 65 [17] and a reduction in consumption of drugs for chronic diseases following the announcement of a future decrease in out-of-pocket prices [18]. We, therefore, conduct analyses trying to separate the effects on utilization due to forward-looking behavior and the pure price effect, respectively.

We find that at the time of the copayment introduction, visits to primary care physician decrease with on average 7%. Our results further show substantial differences across income groups and gender, where women and individuals of low-income households respond stronger to the copayment, 9% and 11%, respectively. Most pronounced effects were found among low-income women. We find no evidence of anticipation effects approaching the copayment threshold.

## Policy context

In the Swedish tax-funded health care system, the responsibility to finance and provide primary and secondary health care lies with each of the 21 regions (county councils); whereas, the 290 municipalities are responsible for the long-term elderly care. Health care costs represent approx. 11% of Sweden’s GDP, which is relatively high compared to other OECD countries [19]. Taxes, set within each region, fund the bulk of health care costs; while, patient out-of-pocket payments and state grants are smaller financing sources [20]. In outpatient primary care, about 40% of physician visits take place with a private provider, and the equivalent number for outpatient specialist care is 25% [21]. The private providers are tax-funded as well, and function under the same policies as the public providers (“patient vouchers”), for example, using identical copayments (although there is also a very small purely private market outside of the tax-funded system).

An outpatient primary care center is the first port of call for non-acute health problems. The primary care centers gather physicians specialized in general medicine, hereafter called primary care physicians, along with nurses, physical therapists, midwives, psychologists, etc. Patients are assigned to a primary care center based on geographic proximity, but are free to switch and register at another center of their choice within the region. The number of physicians is relatively high in Sweden, 4.0 physicians per 1000 inhabitants compared to the OECD average of 3.3 [19]. Despite this, the number of physician consultations in outpatient primary and specialized care is substantially lower in Sweden at 2.9 per capita and year compared to the OECD average of 6.6 [19]. In international comparisons, Swedish health care fares well on most health outcomes such as low levels of infant mortality and high life expectancy, but accessibility and long waiting times are often-debated issues where the health care system fares relatively poorly.

The cost-sharing policy of the Swedish health care system consists of two features—a copayment and a cap (“stop-loss”). Patients are charged a fixed copayment when utilizing health services, and if the total out-of-pocket expenditure reaches the cap within a moving 12-month period, the copayment falls immediately to zero for the remainder of the period. The general principles are the same across the country; while the specific details, for example, the level of copayments are determined within each region.

Our study is set in Region Västra Götaland, one of the larger regions seated in the southwest of Sweden, with 1.6 million inhabitants, out of 10 million inhabitants in total [22]. Compared to other regions and to Sweden as a whole, the region is a “mini-Sweden” with respect to demographic and socio-economic composition and to levels of health care utilization [23]. The copayment for a visit to a primary care physician is about €10 (100 SEK), the copayment for a visit to an outpatient specialist is between €10 and 30, and the 12-month cap for outpatient services is about €110 (1100 SEK). The important feature for our study design is that children and adolescents are excused from copayments up to their 20th birthday. The policy remained the same in the region throughout the period of interest.

## Methods

### Data

We have merged demographic and socioeconomic register data from Statistics Sweden with a regional health care database of all primary care visits. We extracted the data for all individuals born in 1993–1996 that were resident in the region in 2014–2015. The data cover 73,000 individuals of the ages 18–22, with 159,000 visits to primary care physician over 2 years. The Regional Ethics Review Board in Gothenburg approved the merging of the registers and the analysis plan (#359-16). Table A1 in the online appendix gives descriptive statistics for the sample.

The age of the patient at the point of visit determines whether the individual is charged a copayment. Due to confidentiality considerations, the date of birth as provided from the register is given by the quarter of the month. The first quarter relates to days 1–7 of the month, the second to days 8–15, the third to days 16–23, and the fourth to days 24–31, with a total of 48 quarters per year. Age at the point of visit follows the same notation, for example, a person born in the first quarter of July 1995 having a consultation in the fourth quarter of July 2014 will be 19 years and three quarters of a month at the point of visit.

We observe the individuals’ physician visits in each quarter of the month starting from the quarter of the month of the individual’s 19th birthday and up to the quarter of the month of their 21st birthday. This implies 97 quarters in total and a window width of ± 12 months around the age cut-off (threshold). For each specific age, e.g., 19 years and 3 quarters of a month = 19.06 years, we observe the individuals in the sample who pass that age-cell at some point during 2014–2015, and this gives between 35,000 and 38,000 individuals in each age-cell. Table A2 in the online appendix shows a number of background characteristics for the individuals included in a set of selected age-cells. Regressing each of the background characteristics on age shows that they are running smoothly, without discontinuity, across the age threshold (Fig. A1 in the online appendix).

### Regression discontinuity design

We exploit the age discontinuity of the copayment scheme to estimate the effect of copayments on visits to primary care physician using an RD design. If no other variables of importance for the outcome change discretely at the age cut-off and there is no manipulation around the cut-off, we can interpret a discontinuous change in physician visits as a causal effect of the copayment [24, 25]. Using age as the assignment variable in an RD design has the advantage that age cannot be manipulated [see, e.g., 12, 26, 27]. Despite this, age cannot be considered completely randomly assigned because everyone in the sample turns 20 years of age at some point, possibly creating an anticipation effect [24]. In addition, we need to interpret the discontinuity in outcome as the total effect of all factors that discretely change at the age threshold [24]. There are many ongoing lifestyle changes for young adults that might be related to health care utilization, but none that switch on or off at the 20th birthday; rather, they are continuously changing.

We apply a sharp RD because the treatment, i.e., the copayment, is a deterministic function of age. To describe the underlying specification of the RD design, following Lee and Card [28], let $Y_{1}$ and $Y_{0}$ be the potential outcome in physician visits with or without copayment, let $T$ be a binary indicator of treatment, and let $X$ be a running variable of age determining $T$ by a certain threshold $x_{0}$, where $T = 1$ if $X \ge x_{0}$. We collapse the data to a cell-level regression of age-cell means and estimate the following regression equation:

1 $$Y_{j} = \beta_{1} {\text{Treat}}_{j} + h\left( {x_{j} } \right) + \varepsilon_{j} ,$$

where the outcome $Y_{j}$ is the number of visits to primary care physicians per capita and year in age-cell $j$, and $h\left( {x_{j} } \right)$ is a smooth function of age with the specific characteristic of $h\left( {x_{0} } \right) = E\left[ {Y_{0} |X = x_{0} } \right]$. The variable Treat is defined as:

2 $${\text{Treat}} = \left\{ {\begin{array}{*{20}c} {0 \quad {\text{if }}\;x_{j} < x_{0} \;{\text{i}} . {\text{e}} . \; {\text{age}} < 20 } \\ {1\quad {\text{if }}\;x_{j} \ge x_{0} \;{\text{i}} . {\text{e}} . \;{\text{age }} \ge 20} \\ \end{array} } \right. .$$

Our main analysis assesses the effect of copayments on visits to primary care physician. In addition, we compare the main results to copayment effects on visits to outpatient specialists. Thanks to the rich data set, we are able to assess the heterogeneity of the copayment effect and perform subgroup analyses based on gender and income quartile^1^ [29], something few previous studies have done credibly.

For significance testing, we apply conventional robust standard errors and weigh each age-cell by $n_{j} /\left( {N/J} \right)$, where $n_{j}$ is the number of observations in age-cell *j*, *N* is the total number of observations and *J* is the number of age-cells [28]. The cell-level weighted regression gives coefficient estimates equivalent to standard least squares estimates of a micro-level regression [24]. The random specification errors, which are the degree to which the true function $h\left( \cdot \right)$ deviates from the specified polynomial function, are assumed to be independent of treatment status, which implies that the least squares estimate $\hat{\beta }$ is consistent for $\beta_{1}$. The cell-level weighted regression deals with the within-group correlation introduced by these specification errors.

Apart from a linear RD model, we include polynomial functions of the assignment variable to assess the robustness of the treatment effect. However, it should be noted that higher-order polynomials are prone to bias due to noisy estimates and sensitivity to the degree of polynomial [30]. Following Lemieux and Milligan [27] and Bargain and Doorley [26], we consider simple linear, quadratic, and cubic functions and, linear and quadratic splines. We use Akaike’s information criterion (AIC) to assess the goodness of fit of the different model specifications [31]. In addition, we perform a number of alternative estimations to further assess the robustness of the results, with focus on the sensitivity of the bin width of the assignment variable, the width of the window of observations, the use of local linear regression, and the use of false cut-offs. Analysis was performed and graphics created in Stata Statistical Software: Release 15.

### An anticipation effect

The inevitable closing date of free-of-charge health care at age 20 could cause an anticipation effect, such that utilization of health services increases among adolescents approaching the threshold. If so, our model estimates the effect of the threshold rather than the pure price effect. The presence of an anticipation effect is tested by a “donut hole RD” [32], excluding observations ± 1, ± 2, ± 3, and ± 4 months around the threshold. Assessing the copayment effect farther away from the cut-off implies that the samples just below and just above differ slightly in background characteristics, and therefore, we additionally run the test on a restricted subsample where such differences are reduced. We explore the copayment effect in a sample limited to those about 18,000 individuals born between July 1994 and June 1995, and reduce the window width to age-cells 19.5–20.5, which enables to follow this one specific sample across all age-cells. Further, the heterogeneity of the anticipation effect is tested in subgroup analyses based on income quartile and gender.

## Results

### Main results

**Fig. 1:**
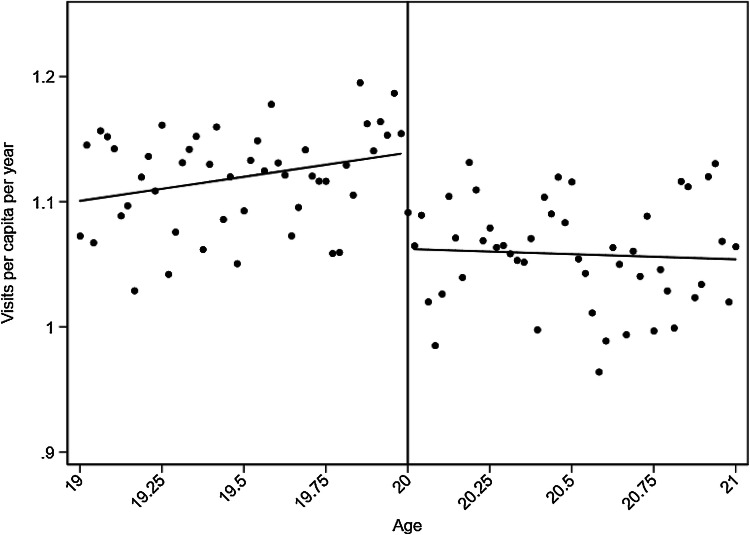
Main results of the estimated copayment effect on visits to physician in primary care. The dots show the mean number of visits at each age, computed by collapsing the individual level data set into age-cell means. The line is the fitted line from running a linear splines regression

Figure 1 graphically shows the results of the main analysis using linear splines. Below the age threshold, the slope is increasing, with a visible downward jump at age 20. This implies that the introduction of the copayment at age 20 reduces the number of primary physician visits.

We estimate a copayment effect of − 0.08 irrespective of model specification (Table A3 in the online appendix) and the results are highly statistically significant with *p* values ≤ 0.001. Goodness of fit by AIC verifies that the specification with linear splines gives the best model fit. With an average of 1.12 visits to primary care physicians per year, the estimated effect corresponds to a 7.1% reduction in visits due to the introduction of the copayment. Table 1 gives the main result as well as the results in the different sub-groups. As a comparison, for visits to outpatient specialists, the copayment effect is estimated to be − 0.02 (statistically insignificant). Specialist visits are also exposed to the copayment introduction at age 20, but presuming a higher severity of disease when visiting a specialist, and considering the more stringent supply rationing in specialized care, we expected to find a smaller copayment effect for physician visits in specialized care compared to visits in primary care.

**Table 1:** Copayment effects and reduction in demand

	Copayment effect (st. err.)	Visits per capita per year, age 19	Reduction in demand (%)	Visits per capita per year, age 20
*Visits to primary care physician*				
All	−0.08 (0.016)***	1.12	− 7.1	1.06
Income quartile				
1st (lowest)	−0.14 (0.035)***	1.21	− 11.4	1.12
2nd	−0.09 (0.031)***	1.12	− 7.9	1.08
3rd	−0.06 (0.033)*	1.10	− 5.5	1.04
4th	−0.02 (0.020)	1.06	− 1.9	1.00
Effect difference between 1st and 4th income quartile	− 0.13 (0.051)**	–	–	
Gender				
Women	−0.13 (0.020)***	1.41	− 9.2	1.32
Men	−0.03 (0.021)	0.86	− 3.5	0.81
Effect difference between women and men	− 0.09 (0.029)***	–	–	
Women in income quartile				
1st	− 0.21 (0.053)***	1.50	− 14.0	1.39
2nd	− 0.14 (0.046)***	1.44	− 9.7	1.35
3rd	− 0.09 (0.044)**	1.38	− 6.5	1.31
4th	− 0.07 (0.054)	1.29	− 5.4	1.23
Men in income quartile				
1st	− 0.09 (0.037)**	0.92	− 9.8	0.82
2nd	− 0.05 (0.036)	0.84	− 6.0	0.81
3rd	− 0.04 (0.040)	0.85	− 4.7	0.81
4th	+ 0.03 (0.042)	0.84	+ 3.6	0.80
*Visits to outpatient specialist*				
All	− 0.02 (0.012)*	0.77	− 2.6	0.76

All estimates from linear splines specifications. *, **, *** corresponding to *p* values ≤ 0.10, ≤ 0.05 and ≤ 0.01, respectively

**Fig. 2:**
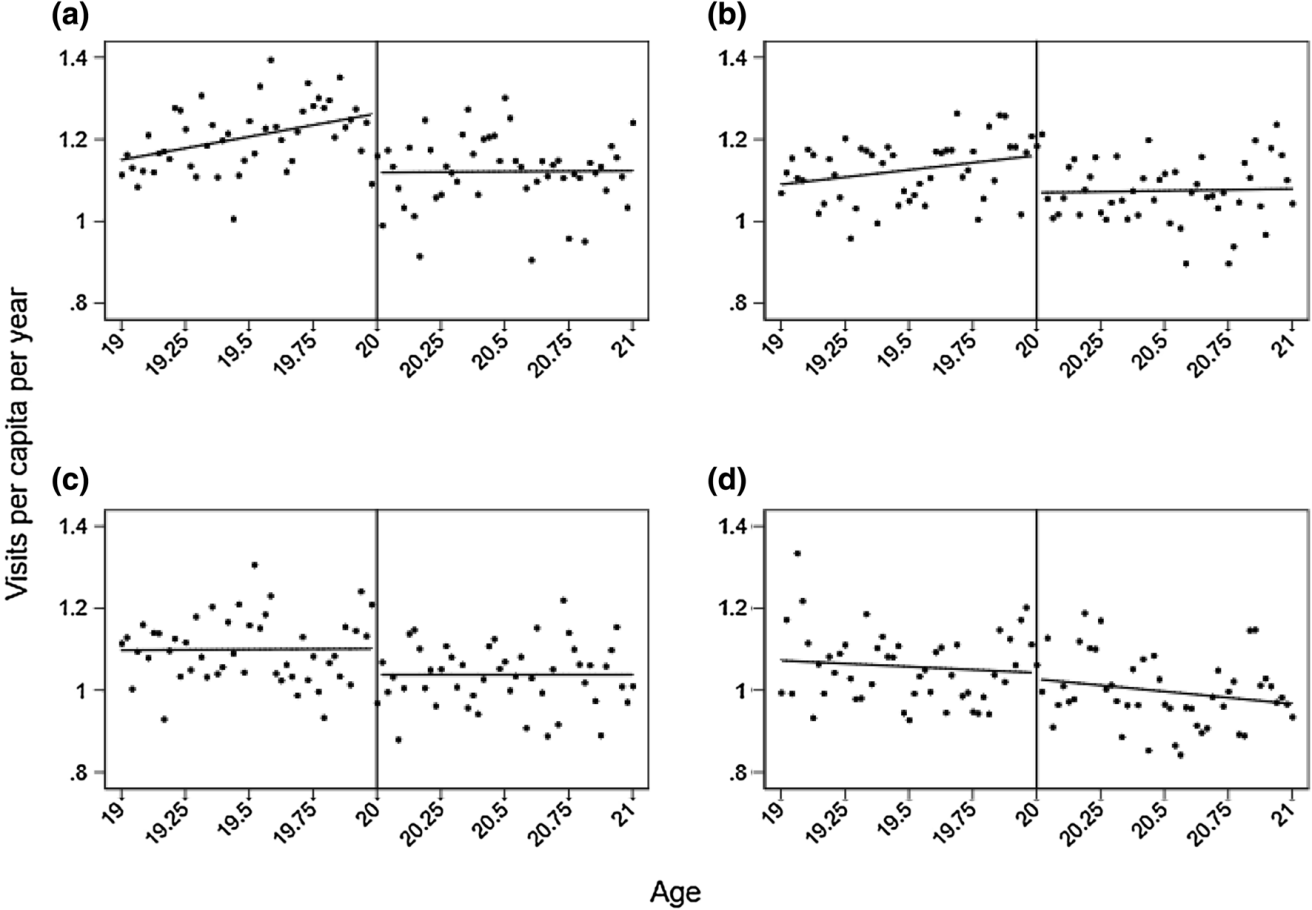
Discrepancies in copayment effects between income groups, for **a** the 1st income quartile (lowest), **b** 2nd income quartile, **c** 3rd income quartile, and **d** 4th income quartile. The dots show the mean number of visits at each age, computed by collapsing the individual level data set into age-cell means for each income group. The lines are the fitted lines from running a linear splines regression for each income group

**Fig. 3:**
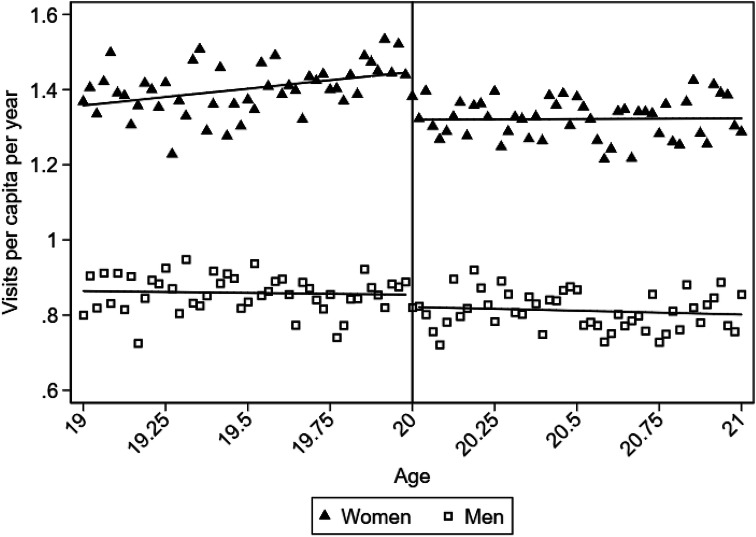
Discrepancies in copayment effects between women and men. The triangles and the squares show the mean number of visits at each age, computed by collapsing the individual level data set into age-cell means for each gender. The lines are the fitted lines from running a linear splines regression for each gender

Assessing the heterogeneity in the price sensitivity, we find a gradient effect of the copayment introduction through income quartiles (Fig. 2; Table 1). The copayment effect estimated by linear splines is − 0.14, − 0.09, − 0.06, and − 0.02 for the 1st, 2nd, 3rd and 4th, income quartiles, respectively (estimates statistically insignificant for the 3rd and 4th income quartiles). The 1st quartile corresponds to the group with the lowest incomes. The copayment causes reductions in demand by 11.4%, 7.9%, 5.5%, and 1.9%, respectively. Hence, there are substantial differences in price sensitivity across income groups. The effect difference between the 1st and the 4th income quartiles is estimated as − 0.13, statistically significant with *p* value < 0.05. With respect to gender, we estimate a notable difference in the copayment effect, − 0.13 for women and − 0.03 for men (estimate statistically insignificant for men). The estimated copayment effect corresponds to a reduction in demand by 9.2% and 3.5% for women and men, respectively (Fig. 3; Table 1). The effect difference between women and men is estimated as − 0.09, statistically significant with *p* value < 0.01.

Separating groups based on both gender and income, we estimate the largest copayment effects (in absolute terms) among women in low-income groups, corresponding to a 14% reduction in demand by women in the 1st income quartile. Among men in the highest income quartile, there is no statistically significant effect. We have also performed subgroup analyses based on other background characteristics, finding a larger copayment effect among those born abroad, those having parents with lower education and those living away from parents, compared to their respective counterparts (Fig. A2 and Table A4 in the online appendix). In subgroups based on income quartile and mentioned background characteristics, the general pattern of larger copayment effects in low-income groups remains. The largest copayment effect (− 0.45) is estimated among individuals in the 1st income quartile living away from their parents. The income gradient in the estimated copayment effect is less clear among those born abroad and those having parents with higher education.

### Robustness checks

Table A5 and Figs. A3–A8 in the online appendix give the results from the robustness checks. Checking the robustness of our results, we first consider the bin width of the assignment variable, i.e., the width of the age-cells, as suggested by Lee and Lemieux [24]. We increase the bin width creating 24 monthly bins, assessing the copayment effect farther away from the cut-off but with less variability in the outcome variable. This yields a copayment effect of − 0.08 to − 0.09 (Fig. A3). Another way to assess the trade-off between precision and bias is to explore the window of observations included [24, 26]. To come closer to the threshold, we reduce the window width in the linear splines specification to ± 9, ± 6, ± 3, and ± 1.5 months around the cut-off. We find that both the size of the copayment effect, estimated between − 0.07 and − 0.16, and the slope of the lines, are sensitive to the range of values being used (Fig. A4).

In a non-parametric local linear regression with a triangular kernel, more emphasis is given to observations close to the threshold [33, 34]. We use a data-driven approach to choose the kernel bandwidth [30, 31], which yields an optimal bandwidth of 11 quarters of the month and an estimated copayment effect of − 0.11 (Figs. A5, A6). We check bandwidth sensitivity by plotting treatment effect estimates as a function of bandwidth [31] and find that the estimated copayment effect is stable in sign and size. We perform the regression using age-cell means and weights like in the main analysis, and as suggested by Calonico et al. [35], we use robust bias-corrected standard errors and *p* values. When using few observations, the functional form is likely to be linear [31], but we also try a local quadratic regression, estimating an copayment effect of − 0.05, however, insignificant.

Knowing the date of birth and the date of visit by the quarter of the month implies that of those paying a visit to a physician in the monthly quarter of their 20th birthday (age-cell 0), some individuals had their visit just the day before their birthday (free of charge) and some just the day after (charged the copayment). In the main estimations, we exclude the quarter of the month of the 20th birthday, creating a small gap at the cut-off. As a robustness check, we provide two different ways to deal with the imprecision of age-cell 0, yielding results ranging from − 0.07 to − 0.08.

A final check of robustness is a set of falsification tests, or “placebo regressions”, of the discontinuity [24, 26, 27]. We run linear splines specifications with a window width of ± 12 months for all possible cut-offs between ages 19.00 and 19.75 years and estimate effects ranging from − 0.01 to + 0.03, almost all of them statistically insignificant (Fig. A7). For all possible cut-offs between 20.25 and 21.00 years, the estimated effects range from − 0.04 to + 0.07. In Fig. A8 in the online appendix, we show the distribution of t-statistics from the falsification tests, the majority estimated between − 2 and 2, while the *t* statistic of the main model specification was − 4.83. Additionally, we check the robustness for the false cut-offs 19.5 and 20.5 years in quadratic splines, increased bin width, and reduced window width, resulting in estimates ranging from − 0.05 to + 0.03, all but two statistically insignificant. We find that even minor changes in model specification lead to a variety of estimates for the false cut-offs, while similar changes do not significantly affect the results at the true threshold. Taken together, the robustness checks yield results in line with the main analysis, estimating copayment effects between − 0.05 and − 0.16, corresponding to a 4.5–14.3% reduction in visits to primary care physicians, while the findings for the false cut-offs do not remain robust.

Running an alternative model specification for the different subgroups, the quadratic splines regressions yield slightly different estimates compared to the linear splines. However, the pattern of larger effects among low-income groups and women remains unchanged.

### Anticipation effect or pure price effect?

Testing the presence of an anticipation effect (results available in Tables A6–A7 and Figs. A9–A10 in the online appendix) by the donut hole RD gives copayment effects between − 0.05 and − 0.07, slightly smaller than our main results but in the range of the results from our robustness checks (Table A6; Fig. A9). Using a restricted sample to account for differences in background characteristics when farther away from the threshold, we estimate a copayment effect of − 0.08 at the threshold, and between − 0.06 and − 0.15 ± 1, ± 2, and ± 3 months away from the threshold; also within the range of our robustness checks (Fig. A10). Hence, we find no evidence of an anticipation effect in the general sample. In an alternative way to deal with discrepancies in background characteristics, we include a set of covariates as control variables in the regressions (Table A7). Assessing the heterogeneity of the price sensitivity, there are indications of an increasing trend among women and lower-income quartiles approaching the threshold, while this pattern was not apparent among men and higher-income quartiles. Testing the anticipation effect among women, we see a pattern of smaller effects (in absolute terms) further away from the threshold, but the difference in estimates is statistically insignificant (Table A6). In sum, the donut hole RD indicates that the main results are driven by actual price effects rather than by anticipation effects.

## Discussion and conclusions

Using an RD design, we have evaluated the effect of the introduction of a copayment at age 20 on the number of primary care physician visits. Our results show a statistically significant decrease by 7% in visits caused by the introduction of the copayment. The results remain robust through various checks yielding results between 5 and 14%. We also found substantial differences across income groups and between women and men. The copayment reduced visits by 9% among women and by 12% among low-income individuals. The most pronounced effects were found among low-income women with a reduction of 14%. In a donut hole RD, we explored the presence of an anticipation effect when approaching the copayment threshold. We find no evidence of statistically significant anticipation effects, although the evidence from our sample indicated slight anticipation effects among women and low-income groups.

**Fig. 4:**
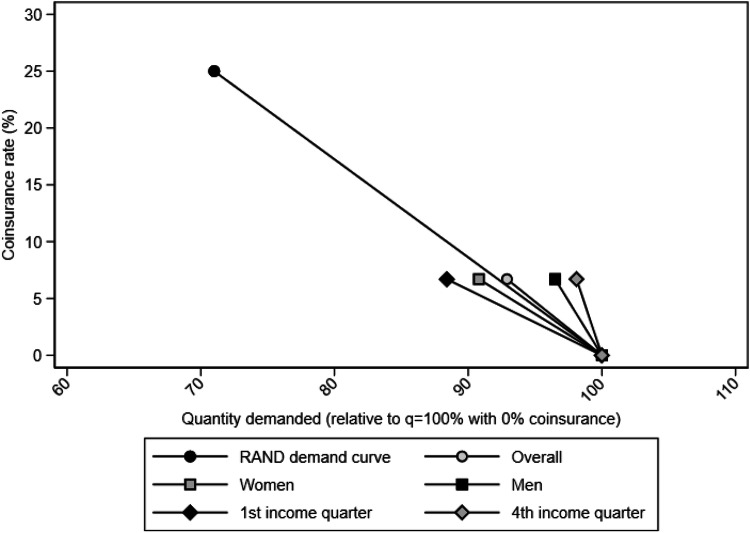
Comparison of demand curves. The Rand demand curve is based on data from Keeler and Rolph [7], who estimated the quantity of outpatient services demanded with a 25% coinsurance rate as the percentage of spending relative to 100% quantity demanded with a 0% coinsurance rate (for chronic episodes, 71% quantity demanded). The demand curves formed by our estimates are under the assumption of an economic cost of a visit to physician in primary care in Sweden being SEK 1500 [38], giving the “coinsurance rate” 100/1500 = 6.67%. Hence, our estimate of a 7.1% reduction in demand due to the copayment, thus, corresponds to 92.9% quantity demanded (relative to 100% quantity demanded with no copayment)

To provide some context, we compare our results to the demand curves as implied by the Rand Health Insurance Experiment (HIE). Using the same approach as in Keeler and Rolph [7], we can estimate and plot demand curves of our results next to the demand curve of the Rand HIE data (Fig. 4). The sample mean copayment effect in our paper yield a demand curve very close to the Rand demand curve. For the subgroup-specific results, we find that the demand curves for men and for high-income individuals are steeper than the Rand demand curve, i.e., less price sensitive in comparison. The demand curves for women and for low-income individuals are flatter, i.e., greater price sensitivity compared to the Rand estimates.

Distributional effects of cost-sharing policies have not been well researched in the previous literature. On the account of the high-quality register data we have used, our study contributes with estimates of heterogeneity in copayment effects based on income and gender, and some additional background covariates. Similar to our findings, Olsen and Melberg [36] found that teenage Norwegian girls had a higher responsiveness to the abolition of copayments for physician visits compared to boys. In contrast to our findings, Cockx and Brasseur [37] found men to be more price sensitive than women in demand for physician services in Belgium. We note that, as expected, there are large discrepancies between the genders in the rate of visits [see, e.g., 23]. There is, however, no intuitive logic behind why men or women would be more price sensitive, and it remains to be explored what mechanisms may cause these differences. A potential mechanism is that those with a higher level of use are more aware of the copayment threshold.

Our study population of young adults includes the entire range of the income distribution, enabling direct comparison between income groups. Our findings that low-income groups are more price sensitive in demand for physician services than high-income groups are in line with assumptions in previous discussions [2], and in agreement with results from Nilsson and Paul [13]. Documenting these findings in a policy context with internationally low-income inequalities, we can potentially see the income gradient in the price effect shown here as a lower bound for countries with higher income inequalities, e.g., the US.

We do not imply that the differences in price effect are caused by income itself, since there are other correlated factors not controlled for. We find in subgroup analyses that, as expected, the differential copayment effects across income quartiles are dependent on gender; parental education; place of birth; and whether or not the youth is living with their parents (i.e., the composition of the household). Young adults of low-income family background are more affected by health care prices, and especially so in combination with being female, being born abroad, having parents with lower education or living away from your parents.

We note that there is an income gradient not only in the estimated price effect, but also in the average number of visits in different income quartiles, with a higher number of visits per capita in low-income groups. The income gradient in visits is only slightly reduced after the copayment introduction. Our income variable is defined as equivalized household income, which implies that the usual gender–income correlation is not as persistent as usual in our study population because 80% of the adolescents lived with their parents; thus, household income is based mainly on parental income.

The RD identification strategy assumes that no other factors change discontinuously at the threshold. Under Swedish regulations, age 18 gives access to alcohol in restaurants and bars, and age 20 permits buying alcohol in liquor stores. Sudden alcohol-related health problems presumably affect emergency visits, while a discontinuous rise in visits to primary care physicians seems questionable. However, in that case, our results can be seen as a conservative estimate of the true copayment effect.

In line with previous literature, we find significant but relatively modest effects of out-of-pocket prices on health care utilization, and we add to the literature credible estimates of heterogeneity in the behavioral responses. Future research should aim to investigate the causal mechanisms of such discrepancies, for example, what grounds lead women and low-income groups to become more price sensitive compared to their respective counterparts.

## Electronic supplementary material

Below is the link to the electronic supplementary material.

Supplementary material 1 (PDF 1010 kb)

## Footnotes

1 Income is measured as the equivalized disposable income of the household, which is calculated taking into account all incomes of the household (family), weighted by the number of individuals in the household and their age. Disposable income includes wages, business profits, transfers, pensions, unemployment insurance payouts, taxes, profits from capital, etc. The sum can be negative. The 1st income quartile corresponds to the group with the lowest incomes.
