# Supplementary material for: Effects of primary care cost-sharing among young adults: varying impact across income groups and gender
Source: Eur J Health Econ. 2019 Aug 10;20(8):1271–80. doi: 10.1007/s10198-019-01095-6 (PMC6803576; doi:10.1007/s10198-019-01095-6)
Supplement: Supplementary file 1 — Supplementary material 1 (PDF 1010 kb) [file 10198_2019_1095_MOESM1_ESM.pdf]

Effects of primary care cost-sharing among young adults: varying impact across income groups and gender

Naimi Johansson, Niklas Jakobsson, Mikael Svensson

Online Appendix

Tables

Table A1 Descriptive statistics of incomes and physician visits in different groups
Table A2 Background statistics for selected age-cells
Table A3 Results and goodness-of-fit from different model specifications
Table A4 Additional results from heterogeneous effects
Table A5 Results from robustness checks
Table A6 Results of tests for an anticipation effect
Table A7 Robustness of anticipation effect, adjusting for background covariates

Figures

Fig. A1 Regression of age-cells on background covariates
Fig. A2 Discrepancies in copayment effects in different subgroups
Fig. A3 Robustness check by changing the bin width
Fig. A4 Robustness check by varying the window width
Fig. A5 Robustness check by local linear and local quadratic regression
Fig. A6 Bandwidth sensitivity of the local regressions
Fig. A7 Estimated effect of falsification tests
Fig. A8 Distribution of t-statistics from falsification tests
Fig. A9 Test of anticipation effect
Fig. A10 Results from estimation using a restricted sample

Tables

Table A1 Descriptive statistics of incomes and physician visits in different groups

	Obs	*Median*	*Mean*	*St. Dev.*	*Min*	*Max*
Income (SEK), 2014						
All	72,882	221,295	239,361	339,389	−2,043,339	78,200,000
1st income quartile (lowest)	18,222	104,127	94,024	49,102	−2,043,339	146,669
2nd income quartile	18,219	186,200	185,300	21,221	146,670	221,293
3rd income quartile	18,221	256,097	256,720	21,273	221,296	295,875
4th income quartile	18,220	355,612	421,413	632,425	295,877	78,200,000
Per capita visits to primary care physician, 2014						
All	72,882	1	1.12	1.58	0	20
Women	35,089	1	1.41	1.76	0	17
Men	37,793	0	0.86	1.33	0	20
1st income quartile	18,222	1	1.21	1.73	0	20
2nd income quartile	18,219	1	1.12	1.57	0	17
3rd income quartile	18,221	1	1.09	1.52	0	17
4th income quartile	18,220	1	1.06	1.48	0	15
Born in Sweden	65,022	1	1.13	1.58	0	20
Born abroad	7,860	1	1.08	1.59	0	14
Having parents with secondary educ. (or lower)	35,992	1	1.17	1.63	0	20
Having parent(s) with tertiary educ.	35,387	1	1.07	1.51	0	15
Living without parents 2014	14,013	1	1.26	1.74	0	20
Living with parent(s) 2014	57,401	1	1.09	1.53	0	17
Per capita visits to outpatient specialists, 2014						
All	72,882	0	0.80	1.83	0	69

Note. SEK 1,000 ≈ $120 (2017). Income is measured as the equivalized disposable income of the individual, which is calculated taking into account all incomes of the household (family), in relation to the number of individuals in the household and their age. Disposable income includes wages, business profits, transfers, pensions, unemployment insurance payouts, taxes, profits from capital, etc. The sum can be negative. (Statistics Sweden, 2016a). The 1st income quartile corresponds to the group with the lowest incomes.

Table A2 Background statistics for selected age-cells

Quarter of the month (from 20th birthday)	*−48*	*−24*	*−16*	*−12*	*−8*	*−1*	*1*	*8*	*12*	*16*	*24*	*48*
Age	19.00	19.50	19.67	19.75	19.83	19.98	20.02	20.17	20.25	20.33	20.50	21.00
Number of individuals in age-cell	34,588	35,267	35,662	35,897	36,124	36,334	36,375	36,706	36,781	36,913	37,292	38,294
Men (%)	52.01	52.11	51.99	52.04	51.92	51.72	51.71	51.76	51.63	51.67	51.60	51.71
Born in Sweden (%)	88.44	88.71	89.01	89.12	89.17	89.26	89.30	89.39	89.47	89.55	89.58	89.92
Having parent(s) with tertiary education (%)	49.55	48.93	48.90	48.79	48.69	48.49	48.48	48.37	48.32	48.22	47.90	47.65
Living with parent(s) at end of 2014 (%)	91.18	86.72	84.97	83.98	83.02	81.40	80.97	79.33	78.34	77.30	74.92	67.54
Median income 2014 (SEK)	227,737	227,242	227,212	226,781	226,417	225,716	225,515	224,317	223,598	222,956	220,810	213,672

Note. Table A2 shows a number of background characteristics for the individuals included in a set of selected age-cells. The first row of the table shows age corresponding to the specific age-cell, and the second row shows the number of individuals in each age-cell. We can see, among the younger individuals in the sample, the share of men is larger, the share born domestically is smaller, a larger share has at least one parent with tertiary education and more of them live with at least one of their parents. Median income is larger for the younger group (because more of them live with their parents). SEK 1,000 ≈ $120 (2017).

Table A3 Results and goodness-of-fit from different model specifications

	Copayment effect (st. err.)	*Goodness-of-fit AIC*
Linear	−0.076 (0.016)	−337.963
Linear splines	−0.077 (0.016)	−338.683
Quadratic	−0.077 (0.016)	−337.498
Quadratic splines	−0.087 (0.023)	−337.137
Cubic	−0.079 (0.022)	−335.512

Note. All estimates are statistically significant with p-values ≤ 0.001.

Table A4 Additional results of heterogeneous effects

	Born in Sweden		*Born abroad*	
All	–0.07 (0.017) ***	65,022	–0.15 (0.039) ***	7,860
Income quartile				
1st (lowest)	–0.13 (0.042) ***	13,162	–0.16 (0.050) ***	5,060
2nd	–0.08 (0.030) ***	16,678	–0.06 (0.109)	1,541
3rd	–0.07 (0.032) **	17,505	–0.09 (0.164)	716
4th	–0.002 (0.037)	17,677	–0.35 (0.183) *	543
	Parental education second.		*Parental education tertiary*	
All	–0.10 (0.021) ***	35,992	–0.04 (0.023) *	35,387
Income quartile				
1st (lowest)	–0.23 (0.050) ***	10,332	–0.003 (0.051)	6,560
2nd	–0.06 (0.033) *	10,525	–0.13 (0.051) **	7,563
3rd	–0.06 (0.050)	9,093	–0.07 (0.046)	9,092
4th	–0.05 (0.069)	6,042	–0.001 (0.040)	12,172
	Living without parents		*Living with parent(s)*	
All	–0.16 (0.039) ***	14,013	–0.06 (0.019) ***	57,401
Income quartile				
1st (lowest)	–0.45 (0.112) ***	9,105	–0.12 (0.040) ***	8,448
2nd	–0.15 (0.069) **	3,626	–0.08 (0.034) **	14,257
3rd	–0.03 (0.035)	1,063	–0.06 (0.035) *	16,906
4th	–0.03 (0.027)	219	–0.003 (0.927)	17,790

Note. Column 2 and 4 give the estimated copayment effect with robust standard errors in parentheses. *, **, *** corresponding to p-values ≤ 0.10, ≤ 0.05 and ≤ 0.01, respectively. Column 3 and 5 give the number of individuals in each group.

Table A5 Results from robustness checks

Bin width: month		*Window width (linear splines)*	
Linear	−0.081 (0.023) ***	±9 months	−0.071 (0.019) ***
Linear splines	−0.082 (0.022) ***	±6 months	−0.102 (0.021) ***
Quadratic	−0.082 (0.023) ***	±3 months	−0.156 (0.029) ***
Quadratic splines	−0.095 (0.033) ***	±1.5 months	−0.118 (0.041) **
Cubic	−0.086 (0.035) **		
Non-parametric local regression			
Linear splines	−0.114 (0.026) ***		
Quadratic splines	−0.049 (0.031)		
Imprecision of age-cell 0	$T = 1\ if\ age \geq 20$	Imputed age-cell –0.5	
Linear	−0.073 (0.016) ***	−0.070 (0.016) ***	
Linear splines	−0.075 (0.015) ***	−0.071 (0.016) ***	
Quadratic	−0.074 (0.016) ***	−0.071 (0.016) ***	
Quadratic splines	−0.083 (0.021) ***	−0.072 (0.024) ***	
Cubic	−0.073 (0.021) ***	−0.068 (0.023) ***	
Falsification tests	Cut-off 19.5	Cut-off 20.5	
Bin width: quarter of month			
Linear splines	0.028 (0.015)*	−0.023 (0.017)	
Quadratic splines	0.032 (0.023)	−0.036 (0.029)	
Bin width: month			
Linear splines	0.022 (0.016)	−0.012 (0.021)	
Quadratic splines	0.007 (0.015)	−0.003 (0.043)	
Window width: ±6 months			
Linear splines	0.008 (0.021)	−0.051 (0.024)**	
Quadratic splines	0.034 (0.033)	−0.043 (0.035)	
Heterogeneity (quadratic splines)			
1st income quartile	−0.152 (0.054) ***	Women	−0.157 (0.030) ***
2nd income quartile	−0.075 (0.049)	Men	−0.021 (0.031)
3rd income quartile	−0.073 (0.052)		
4th income quartile	−0.047 (0.052)		

Note. Each cell gives the estimated copayment effect with robust standard errors in parentheses. *, **, *** corresponding to p-values ≤ 0.10, ≤ 0.05 and ≤ 0.01, respectively. Regarding the imprecision of age-cell 0, we try two alternative regressions. First, we assume that all patients paying visits in age-cell 0 are charged a copayment. Second, we use age in years as noted by the care provider at the point of visit to determine the approximate number of visits within the quarter of the month of the 20th birthday that were made just before or just after the birthday. We impute an additional age-cell between −1 and 0, at approximate age 19.99, that includes visits by patients who were reported by the care provider to be 19 years, and we keep the visits where patients were reported to be 20 years in age-cell 0.

Table A6 Results of tests for an anticipation effect

	Main sample	*Restricted sample*	*Women*	*Men*	*1st income quartile*	*2nd income quartile*	*3rd income quartile*	*4th income quartile*
At the cut-off	−0.077<br>(0.016)	–0.080<br>(0.029)	−0.127<br>(0.020)	−0.033<br>(0.021)	−0.143<br>(0.035)	−0.091<br>(0.031)	−0.063<br>(0.033)	−0.015<br>(0.037)
Away from the cut-off by ±1 month	−0.063<br>(0.016)	–0.060<br>(0.029)	−0.101<br>(0.019)	−0.031<br>(0.021)	−0.150<br>(0.033)	−0.093<br>(0.028)	−0.037<br>(0.031)	+0.016<br>(0.032)
±2 months	−0.054<br>(0.015)	–0.066<br>(0.033)	−0.093<br>(0.021)	−0.022<br>(0.020)	−0.123<br>(0.035)	−0.075<br>(0.030)	−0.056<br>(0.030)	+0.026<br>(0.033)
±3 months	−0.073<br>(0.014)	–0.152<br>(0.033)	−0.084<br>(0.024)	−0.068<br>(0.019)	−0.088<br>(0.034)	−0.086<br>(0.028)	−0.062<br>(0.031)	−0.067<br>(0.032)
±4 months	−0.074<br>(0.016)	..	−0.071<br>(0.024)	−0.081<br>(0.019)	−0.042<br>(0.035)	−0.067<br>(0.028)	−0.107<br>(0.032)	−0.088<br>(0.031)

Note. All regressions are estimated using a linear splines model specification. Each cell gives the estimated copayment effect with robust standard errors in parentheses. Estimates using the main sample (column 2) are statistically significant with p-values ≤ 0.001. Regressions using the restricted (born July 1994 to June 1995) sample (column 3) are based on a reduced window width from age 19.5 to 20.5 year, and estimates are statistically significant with p-values ≤ 0.05. In subgroup regressions (column 4–9), estimates for women, low-income groups and for all groups ±3 and ±4 months away from cut-off are statistically significant with p-values ≤ 0.05.

Table A7 Robustness of anticipation effect, adjusting for background covariates

	Adjusting for *% men*	*% born in Sweden*	*% having parent(s) with tertiary educ.*	*% living with parent(s)*	*Income*
At the cut-off	−0.062 (0.036)	−0.115 (0.049)	−0.067 (0.071)	−0.077 (0.086)	−0.078 (0.035)
Away from the cut-off by					
±1 month	−0.069 (0.027)	−0.068 (0.020)	−0.078 (0.031)	−0.088 (0.051)	−0.090 (0.042)
±2 months	−0.007 (0.033)	−0.052 (0.024)	−0.043 (0.045)	−0.0002 (0.088)	+0.030 (0.100)
±3 months	−0.072 (0.041)	−0.092 (0.029)	−0.054 (0.051)	−0.067 (0.095)	−0.043 (0.105)
±4 months	−0.067 (0.037)	−0.106 (0.054)	−0.051 (0.067)	−0.068 (0.104)	−0.062 (0.080)

Note. An alternative way to deal with discrepancies between age-cells is to take a closer look on the background covariates in these age-cells and include them as control variables in the regression. Examining some important background characteristics in the samples before the threshold, the share of men is larger; the share born abroad is larger; the share having parent(s) with tertiary education is larger; the share living with parent(s) is larger; and the median income is larger (Figure A1 and Table A2). All these characteristics are associated with a lower level of physician visits compared to their counterparts (Table A1), which would imply under-estimated effects in unadjusted regressions. Due to high correlation between background covariates and age, we perform one regression for each covariate, including the covariate and its interaction with the treatment dummy in a linear splines model specification. In these regressions, we calculate the copayment effect and its standard error at the threshold using the X-value of that age-cell. The copayment effect at the cut-off is estimated between −0.06 and −0.12, however not statistically significant in all cases. ±1, ±2, ±3 and ±4 months away from the cut-off yields varying results, but most estimates are not statistically significant different from our main estimate and within the range of our previous robustness checks, thus no evidence of an anticipation effect. Among these estimates, many are statistically insignificant.

Figures

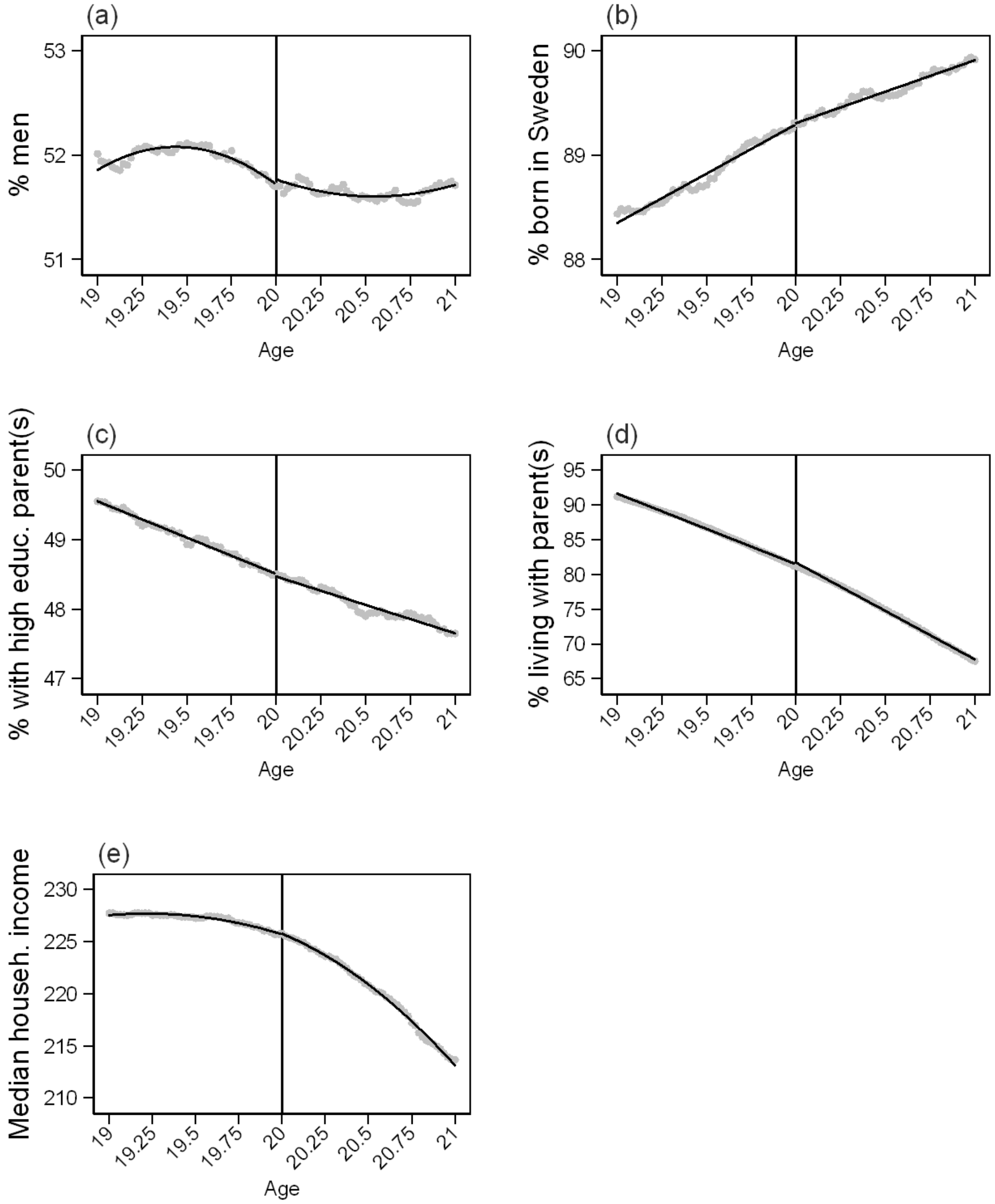

Fig. A1 Regression of age-cells on background covariates

Note. Independent variables being a) percentage of men, b) percentage of individuals born in Sweden, c) percentage having at least one parent with tertiary education, d) percentage living with at least one parent 2014, and e) median household income year 2014.

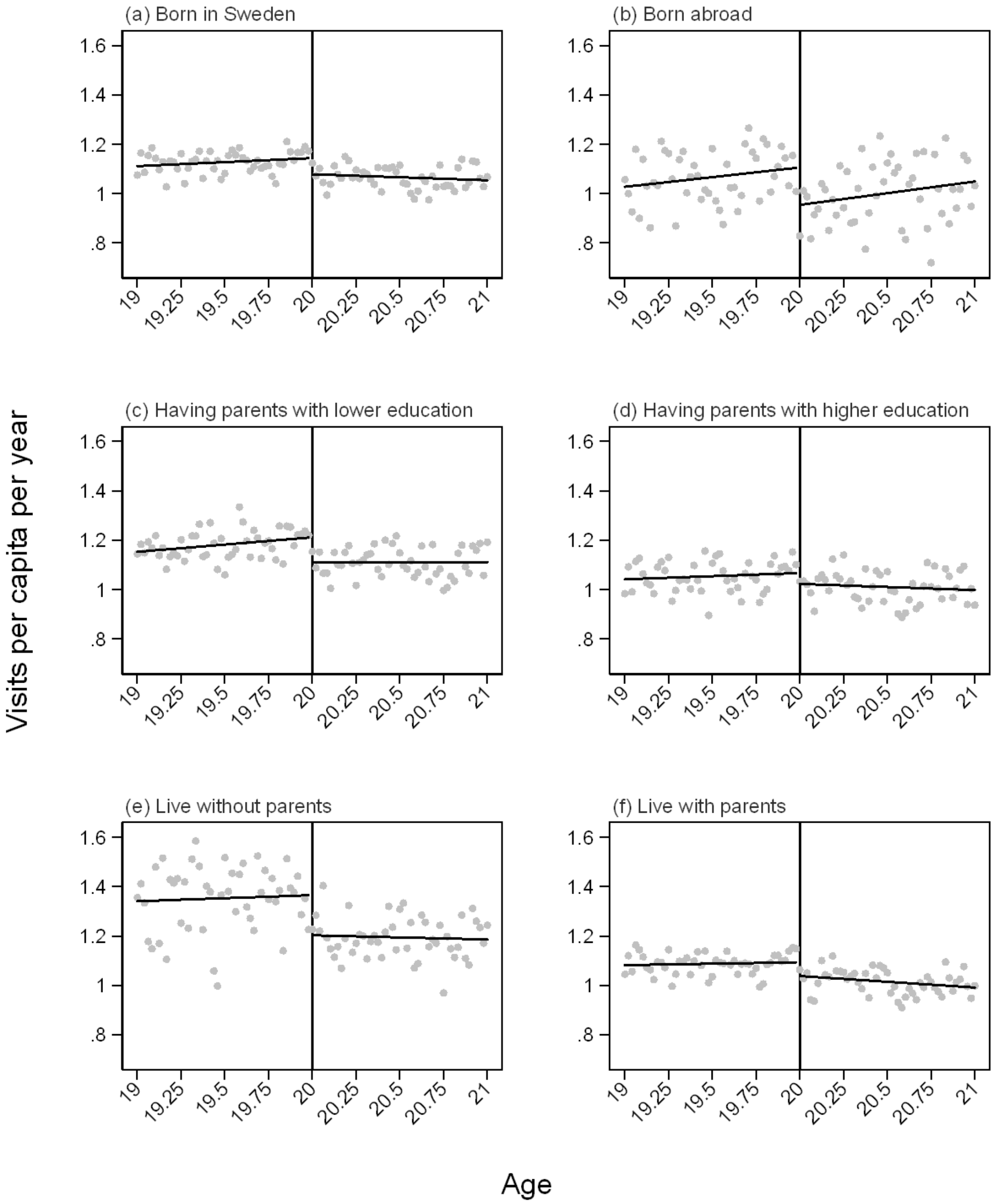

Fig. A2 Discrepancies in copayment effects in different groups

Note. Groups of a) born in Sweden, b) born abroad, c) having parents with secondary education (or lower), d) having parent(s) with tertiary education, e) living without parents, and f) living with parent(s). We have divided the full sample based on birthplace, parental education or living with/without parents, and estimated the copayment effect in each group using the linear splines model specification.

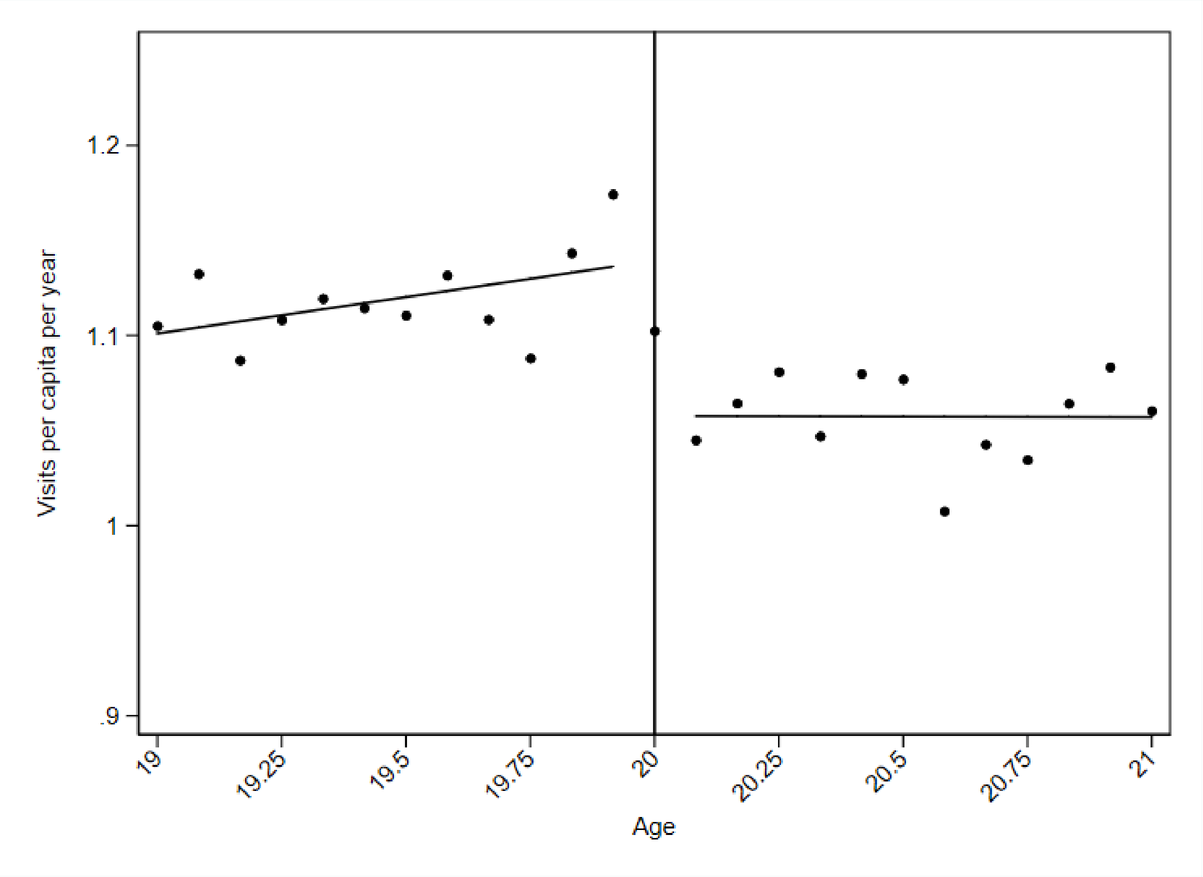

Fig. A3 Robustness check by changing the bin width

Note. In the main specification, we used bin width of a quarter of a month. The narrower the bin, the closer to the cut-off, but with more variation and potentially unnecessary noise. Here in Figure A1, we have used monthly bins in the linear splines model specification. The number of observations is reduced, but still evident is the discontinuity of the estimated regression line at the copayment threshold.

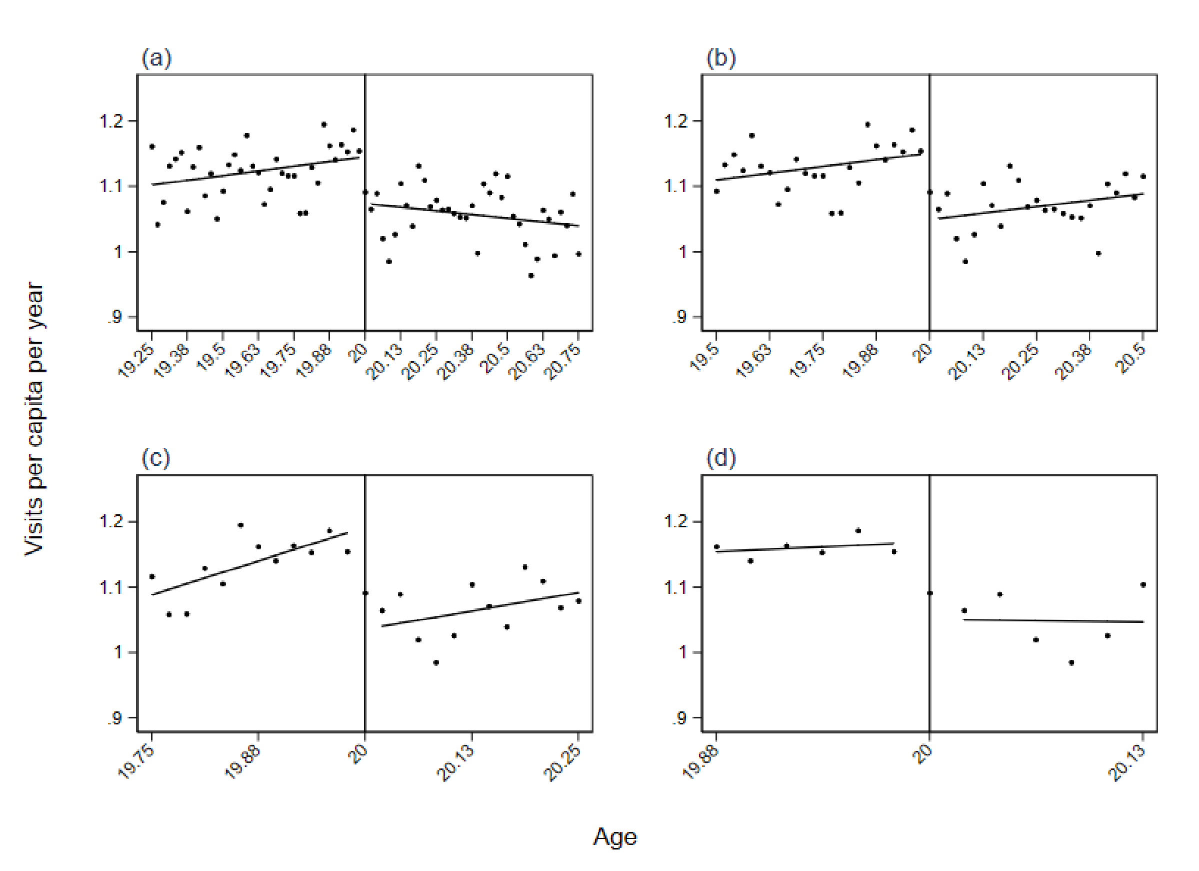

Fig. A4 Robustness check by varying the window width

Note. Width of a) ±9 months, b) ±6 months, c) ±3 months, and d) ±1.5 months around the cut-off. In the main specification, the window width was ±12 months around the cut-off. A larger window of values will give more precise estimates due to a larger number of observations, but a smaller window will reduce the bias of the estimated treatment effect. As seen, using the linear splines model specification, the reduced window width yields varying results of the estimated copayment effect, and varying slopes of the estimated regression lines on both side of the threshold.

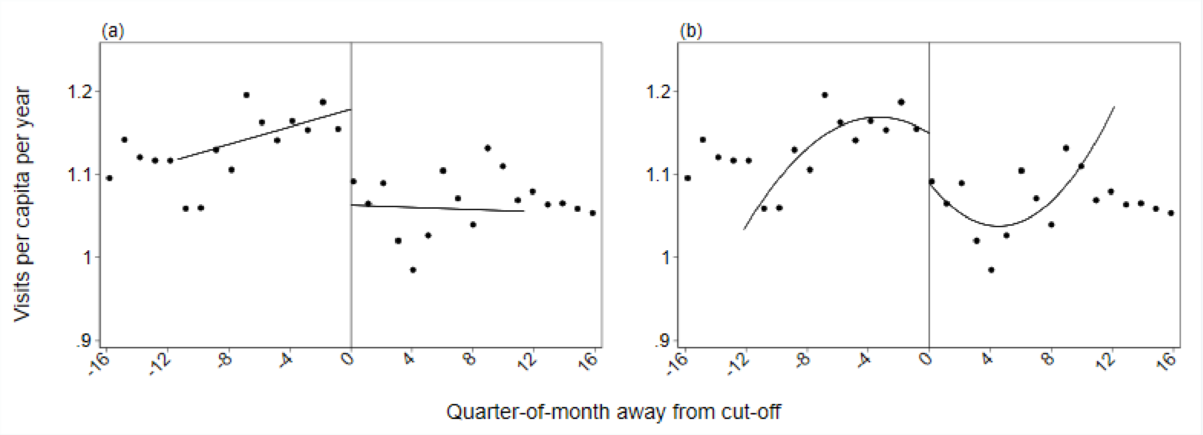

Fig. A5 Robustness check by local linear and local quadratic regression

Note. Local a) linear regression and b) quadratic regression. The local regression is a non-parametric approach where the choice of kernel bandwidth (the window width) is data-driven. The optimal bandwidth is selected to about ±11–12 quarters of a month around the cut-off, corresponding to ±3 months around the cut-off. The local quadratic regression should be interpreted with caution as the polynomial form may be overfitted in such small bandwidth.

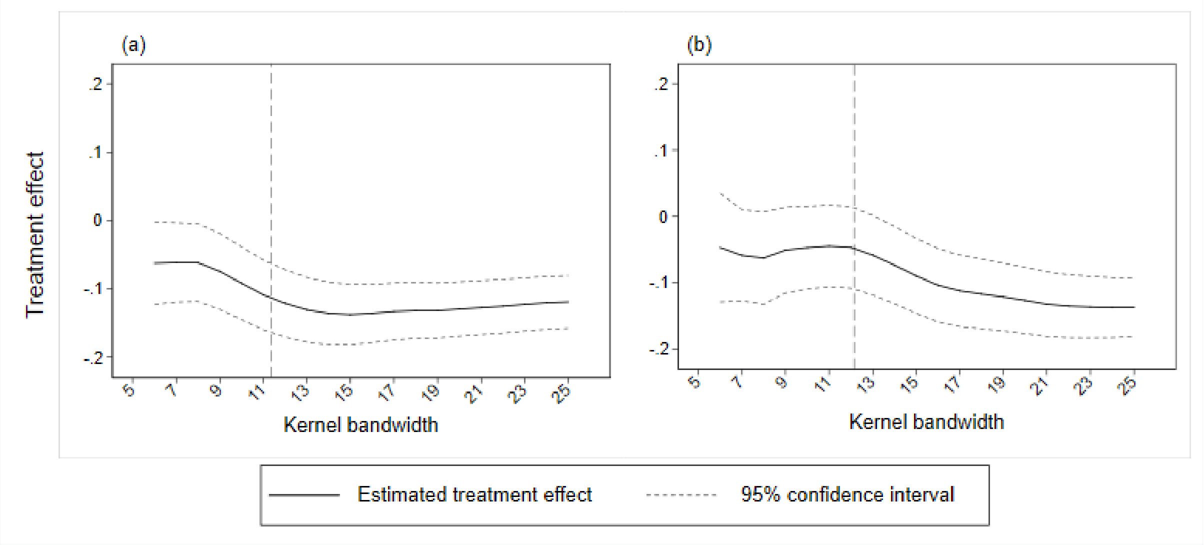

Fig. A6 Bandwidth sensitivity of the local regressions

Note. Local a) linear regression and b) quadratic regression. The optimal bandwidth is marked by the vertical, dashed line. The choice of the kernel bandwidth is data-driven with aims to find a bandwidth where the estimated coefficients are consistent. The local linear regression has found the optimal bandwidth at ±11 quarter of the month from the cut-off, and selecting a somewhat smaller or larger bandwidth yield about the same result, with slightly higher values for a very small bandwidth, but still statistically significant. The results from the local quadratic regression are similar but non-significant for bandwidths about ±12 and smaller.

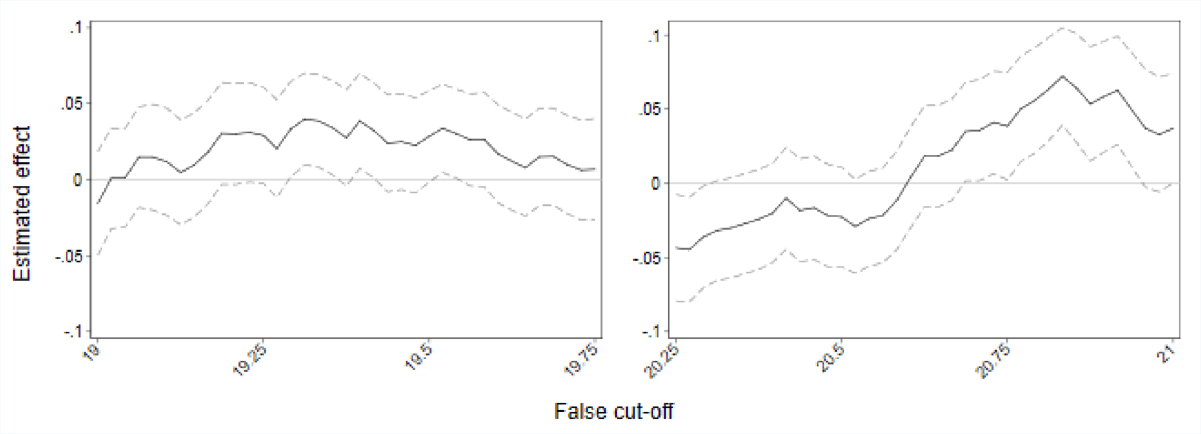

Fig. A7 Estimated effect of falsification tests

Note. Falsification tests given all possible false cut-offs from 19.00 to 19.75 years (left) and 20.25 to 21.00 years (right). The figures show the estimated effect along with 95% confidence interval. In the falsification tests we created false thresholds (all possible) and estimated the threshold effect at each of these threshold, using the linear splines model specification. Possible cut-offs are given by the quarter of month age-cells described in the Data section, i.e. cut-offs at 19.00, 19.02, …, 19.73, 19.75, and 20.25, 20.27, …, 20.98, 21.00. For each threshold, we used a window of ±12 months (e.g. a window between 18.0 years and 20.0 years for a cut-off at 19.0 years). A limitation of these falsification tests is that the true threshold is included in the window for each estimate.

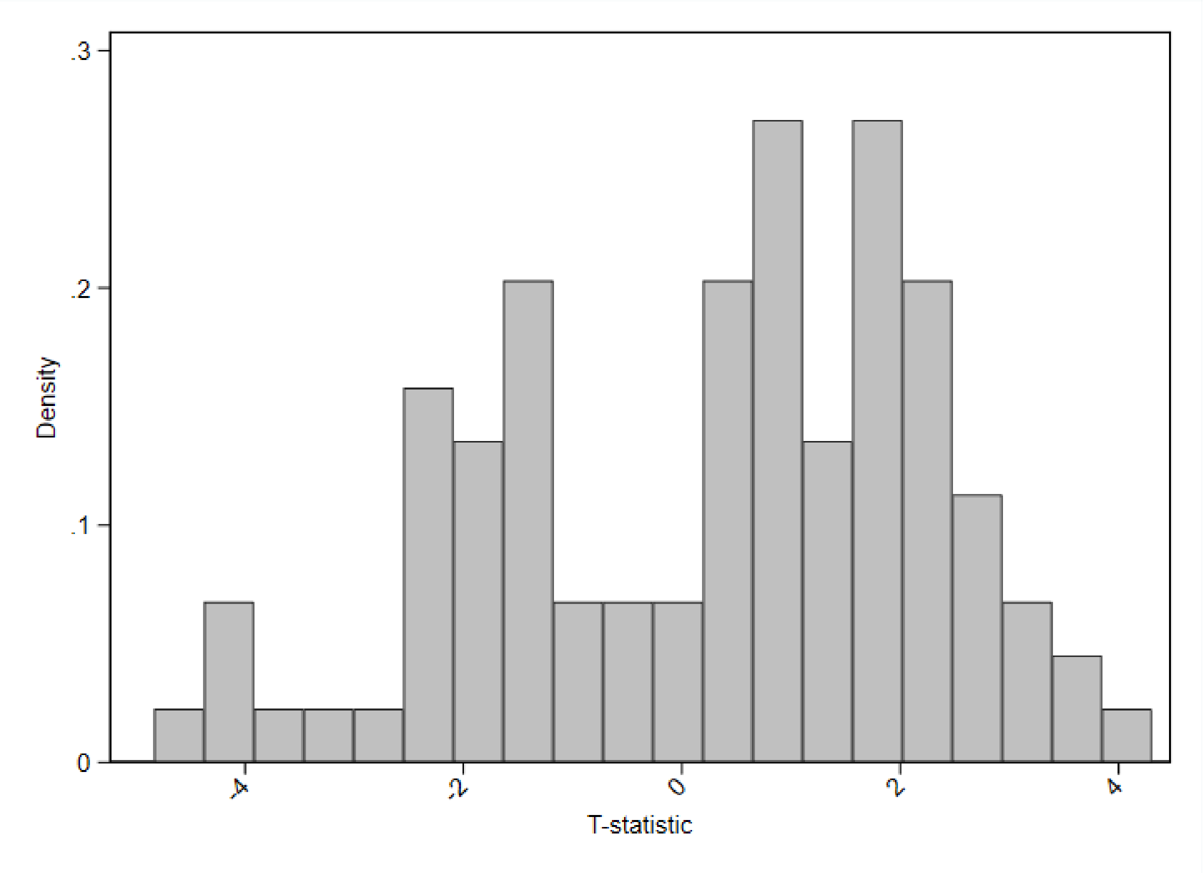

Fig. A8 Distribution of t-statistics from falsification tests

Note. In the falsification tests we created false thresholds (all possible) and estimated the threshold effect at each of these threshold, using the linear splines model specification.

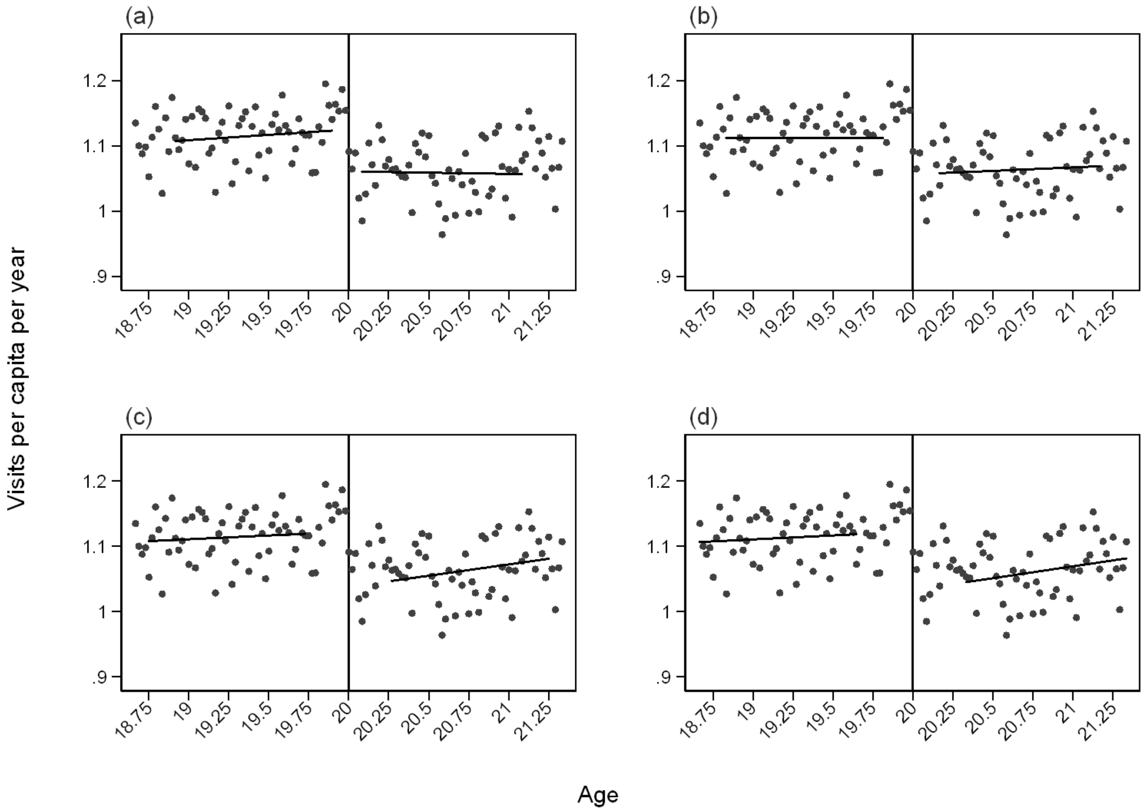

Fig. A9 Test of anticipation effect

Note. Gaps around the threshold of a) ±1 month, b) ±2 months, c) ±3 months, and d) ±4 months.

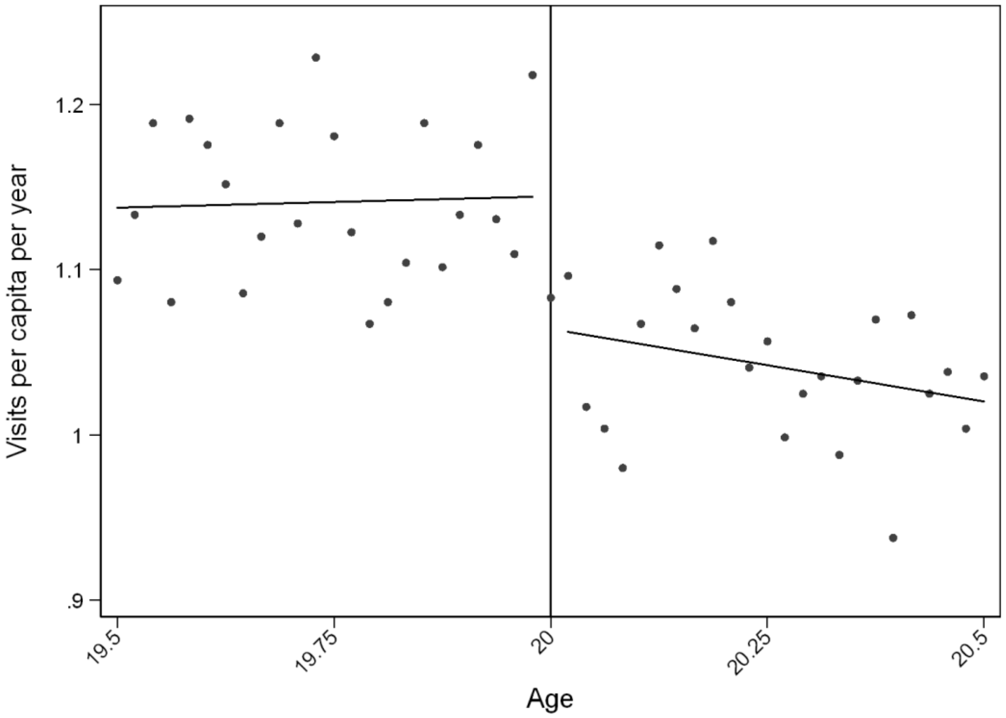

Fig. A10 Results from estimation using a restricted sample

Note. In the restricted sample estimation we have used a subsample of 18,169 individuals born July 1994 through June 1995. Using this subsample makes it possible to follow the same set of individuals over all age-cells through 19.5–20.5, still running over 2014–15. We cannot increase the window width in this case, because some of those born in July 1994 where 19.5 right in the beginning of 2014, and thus 19.4 in the end of 2013, and vice versa for those born in June 1995. So when using the same sample over all age-cells, the window width is restricted.
